# DARIP Technique: A Novel Approach in the Surgical Management of Placenta Accreta Spectrum in the Second Trimester From Tu Du Hospital, Vietnam

**DOI:** 10.1155/crog/7907465

**Published:** 2026-06-08

**Authors:** Ngoc Hai Tran, Phuc Nhon Nguyen, Van Hoang Bui

**Affiliations:** ^1^ Tu Du Clinical Research Center (TD-CRC), Tu Du Hospital, Ho Chi Minh City, Vietnam; ^2^ Department of Pathology Pregnancy, Tu Du Hospital, Ho Chi Minh City, Vietnam; ^3^ Integrated Planning Room, Tu Du Hospital, Ho Chi Minh City, Vietnam

**Keywords:** cesarean section, conservative surgery, placenta accreta spectrum, pregnancy, second trimester

## Abstract

**Background:**

Placenta accreta spectrum (PAS) is an increasingly common complication during pregnancy that has led to high maternal mortality and morbidity in the past decades. Treatment of PAS in the second‐trimester pregnancy includes expectant management and surgical intervention. However, PAS surgery is related to complex obstetric management involving psychological issues and life‐threatening hemorrhage. In low‐ and middle‐income countries, cesarean delivery, along with uterine‐sparing surgery in pregnancies complicated by PAS disorders, could be an optimal choice due to limited resources.

**Methods:**

Herein, we describe a novel procedure in managing PAS in the second trimester of gestation through a clinical case and presentation of a video.

**Case Presentation and Results:**

The 35‐year‐old pregnant woman at term of 21 weeks and 6 days was hospitalized due to important vaginal bleeding. An ultrasound scan revealed PAS type percreta. The patient was managed with the DARIP procedure. The surgical approach included five steps: (1) *D*issection of the uterovesical interface containing newly proliferative vessels, (2) *A*mniotic fluid reduction, (3) *R*ectangular‐shaped hemostatic sutures, (4) *I*ncision of the uterine layer through the vascular‐blocked region, and (5) *P*recise suturing of the placental bed. Regarding surgical outcomes, the estimated blood loss was 300 mL, and no surgical complication was noted. The patient was sent home on Day 7.

**Conclusions:**

The DARIP procedure is a promising method in managing PAS disorders in the second trimester. However, the DARIP technique requires further validation in larger studies. Moreover, this feasible procedure should be performed at an expert center to optimize the maternal outcomes.

## 1. Introduction

Placenta accreta spectrum (PAS) is a placental‐related disorder, relating to morbidly abnormal placental adhesion with an increasing rate of cesarean delivery during the last decade worldwide [1]. According to recent studies, the average incidence of PAS is approximately 0.17%, equal to 1–2 cases per 1000 deliveries and increasing in high‐risk populations with multiple cesarean deliveries and placenta previa [2]. Currently, along with the advanced technology of imaging diagnosis, PAS could be detected early from 14 weeks of gestation [2]. However, an accurate diagnosis and an appropriate management remain a controversial issue. In many cases, the diagnosis of PAS was delayed, and the treatment became more challenging [3]. Although conservative management could be considered, spontaneous abortion of PAS could result in massive vaginal bleeding, hypovolemic shock, and emergency hysterectomy in severe cases [4, 5].

In general, PAS management in the second trimester includes expectant management or termination of pregnancy. The choice of treatment should be discussed with the patient′s desire for fertility preservation and the resources of multidisciplinary management at the expertise center [6]. Expectant management could lead to severe risks such as uterine rupture and massive hemorrhage [7]. Termination of pregnancy could be performed with surgical intervention with the placenta in situ. After cesarean, the patient could be treated by adjuvant therapy, including methotrexate (MTX) or uterine artery embolization (UAE) [8]. However, in low‐resource settings, UAE is unavailable. Monitoring and adjuvant treatment with MTX could lead to severe bleeding and an increased risk of peripartum infection [6].

In addition, cesarean delivery in the second trimester of PAS pregnancy could result in hysterectomy, leading to the irreversible consequence of infertility [9]. Compared with third‐trimester cesarean hysterectomy, second‐trimester gravid hysterectomy demonstrated higher incidences of hemorrhage, blood transfusion, shock, coagulopathy, and hospitalization ≥ 7 days [10]. Therefore, surgical management remains a worsening issue worldwide, especially in centers with low‐resource settings with a high incidence of PAS [11]. At our center, the uterine‐sparing surgical approach relating to the management of PAS in the third‐trimester pregnancies has been recently documented [12–14]. However, the surgical approaches of PAS in the second trimester of gestation remain limited. This report is aimed at describing the novel surgical procedure in managing PAS in the second trimester and reporting a clinical case at our consultant hospital.

## 2. Case Presentation

A 35‐year‐old Vietnamese pregnant woman was hospitalized due to severe vaginal bleeding. The patient had no medical history. She was noted to have a history of cesarean section. The patient did not follow up on her pregnancy at our hospital; thus, the first‐trimester record was missed, and the diagnosis of cesarean scar pregnancy could not be monitored.

**Figure 1:**
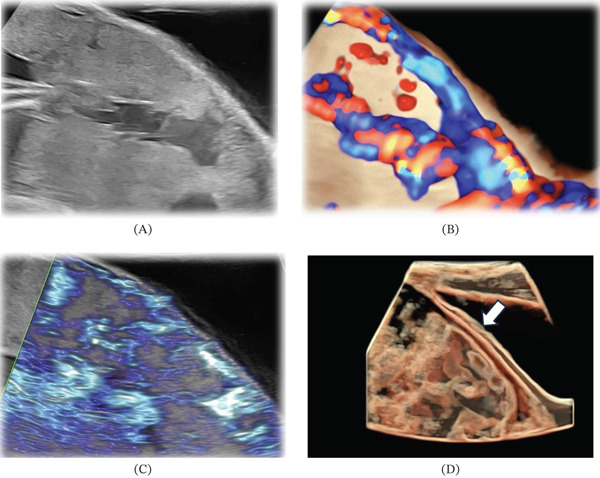
Ultrasound scan shows the placenta located at the anterior wall of the lower uterine segment, covering the internal os of the cervix. (A) Gray‐scale ultrasound signs reveal placental bulge, myometrial thinning, loss of “clear zone,” abnormal lacunae, and bladder wall interruption. Neovascularization signs, such as bridging vessels and “rail signs,” are detected with (B) color Doppler ultrasound and (C) power Doppler ultrasound. (D) Tramline disruption (white arrow) is observed on three‐dimensional ultrasound.

An ultrasound scan found a placenta previa located at the anterior wall of the uterus. In addition, an ultrasound scan revealed ultrasonic features such as loss of clear zone, abnormal placentae lacunae, bladder wall interruption, and myometrial thinning of less than 2 mm. Color Doppler US showed uterovesical hypervascularity—color, subplacental hypervascularity, bridging vessels, and placental lacunae feeder vessels—which suggested a PAS implemented by type percreta at 21 weeks and 6 days (Figure 1A–D and Supporting Information 1: Video S1, Supporting Information 2: Video S2, Supporting Information 3: Video S3, and Supporting Information 4: Video S4). The patient was counseled for termination of pregnancy and was informed of the high risk of massive intraoperative bleeding. Additionally, the risk of cesarean hysterectomy was discussed. However, the patient desired a subsequent pregnancy and would like to preserve her fertility.

**Figure 2:**
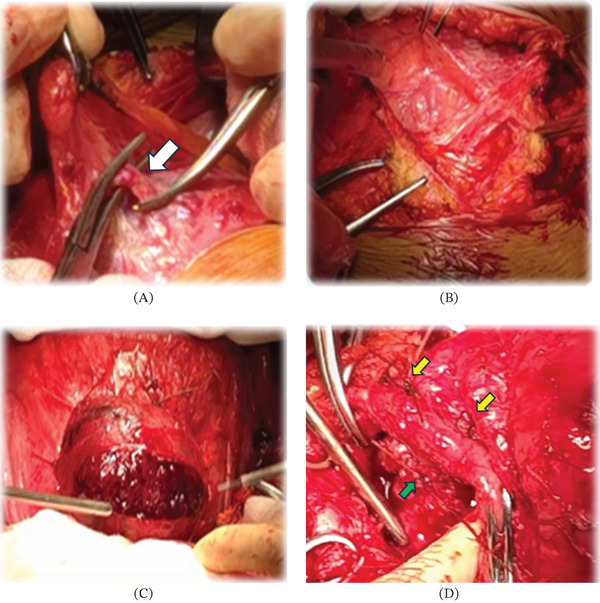
(A) Intraoperative photos demonstrate the presence of newly proliferative vessels (white arrow) at the lower uterine segment and between the anterior uterine wall and posterior bladder wall. (B) The vesicouterine interface was dissected to evaluate the placental invasion into the lower uterine segment. (C) Uterine incision at the placental location following rectangular‐shaped sutures. (D) Placental bed suture (green arrow) and the removal of rectangular‐shaped sutures (yellow arrows) and placental tissue–contained myometrial resection were performed before uterine closure.

Upon laparotomy, the newly proliferative vessels were observed at the lower uterine segment (LUS). The vascular proliferation invaded the serosal layer of the uterus and the uterus–bladder interface. Therefore, the PAS Grade 3c following the International Federation of Gynecology and Obstetrics (FIGO) classification was determined [15]. After uterovesical dissection, the area of anterior placental implantation without involving the cervix was observed (Figure 1B). The mostly upper (Sector S1) part and partly upper (Sector S2) part of the LUS were evaluated and classified as Type 3 following the topographical classification system [16]. The patient underwent the DARIP procedure for conservative uterine surgery (Figure 2A–D and Supporting Information 5: Video S5).

In the DARIP technique, before assessing the surgical procedure, the location of PAS was confirmed by an ultrasound scan. The team sutured the marginal border of the placenta and the invasive myometrial layer of the uterus by using Chromic 1/0 absorbable suture. The suture zone was similar to a rectangular shape. This suture was performed carefully before the uterine incision. All rectangular‐shaped sutures were inserted into the avascular zone of the uterine myometrial layer. This step aimed to avoid bleeding before the uterine incision. After the suture was completely tightened, the neovascularization at the adherent placenta was blocked. Therefore, the purpose of this technique was to reduce the bleeding following the uterine incision. The rectangular‐shaped sutures were removed after placental delivery, followed by excision of the invaded myometrial tissue. The suturing needle penetrated deeply through the myometrial layer where the placenta is located (reaching the maximum thickness of the uterine myometrial layer). Since the newborn could not be viable, the team allowed the needle to touch the amniotic membrane and the parts of the fetus if presented during the surgical intervention.

**Figure 3:**
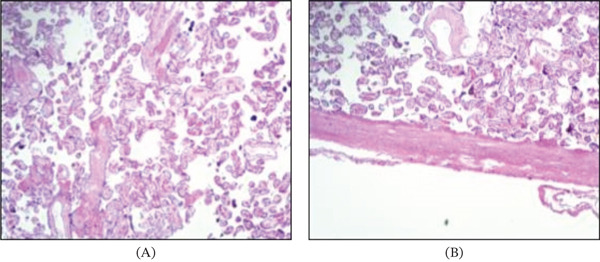
Histopathological examination shows chorionic villi focally invading the myometrium corresponding to the presence of placenta accreta spectrum on hematoxylin and eosin stain (A) x10and (B) x40.

Total intraoperative blood loss was 300 mL. Histopathological examination confirmed a PAS type percreta (Figure 3A,B). The patient recovered uneventfully and was discharged home on the 7^th^ day after surgery. On the follow‐up, no postoperative complication was reported. Her menstrual status was normal. The pregnancy outcome and long‐term outcomes were monitored. The patient and her family were thankful to our team for saving her life.

## 3. Discussion

In the present case, the diagnostic criteria of PAS were confirmed by ultrasound scan, intraoperative evaluation, and histological examination [15]. More than 14 weeks of gestation, diagnosis of PAS disorders could be suspected by ultrasound among patients with a prior cesarean scar [17]. However, the presence of PAS disorders on an unscarred uterus should also be carefully considered to avoid misdiagnosing patients [18]. In challenging cases, the role of magnetic resonance imaging (MRI) could be added to an ultrasound scan [19]. In our case, the diagnosis of PAS by ultrasound was clearly identified. Moreover, the patient was in an emergency condition with severe vaginal bleeding, and the investigation by MRI was not performed.

Since the patient was hospitalized at term of 21 weeks and 6 days, the diagnosis of CSP progressive to PAS could not be identified during the first and second trimesters of pregnancy. In general, 80% of expectant management patients in CSP cases had a PAS [20]. In addition, expectant management was associated with a significantly high complication risk, such as bladder injury, massive bleeding, intensive care unit (ICU) admission, and adverse neonatal outcomes [21].

**Figure 4:**
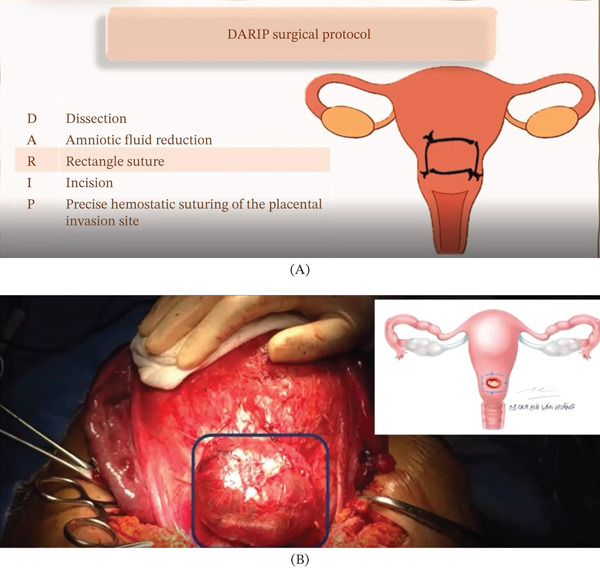
The (A) DARIP procedure and (B) rectangular‐shaped hemostatic sutures.

Regardless of uterus preservation, hysterotomy is a safe choice for terminating a second‐trimester PAS pregnancy [22]. In addition, focal resection could be considered [20]. Our surgical technique was applied for the clinical management of PAS under 22 weeks of gestation. The DARIP technique is an abbreviation word that is written by the first letter of five steps consisting of *D*issection of the uterovesical interface containing newly proliferative vessels, *A*mniotic fluid reduction, *R*ectangular‐shaped hemostatic sutures, *I*ncision of the uterine layer through the vascular‐blocked region, and *P*recise suturing of the placental bed (uteroplacental interface) (Figure 4A,B and Supporting Information 1: Video S1).

In our technique, uterovesical dissection helps in evaluating the placental invasion. Importantly, an amniotic fluid reduction under ultrasound guidance helps in decreasing the size of the uterus and facilitates the exteriorization of the uterus from a regular Pfannenstiel incision. The purpose of amniotic fluid reduction is also for easy manipulation, release of vascular compression, facilitating blood control, and avoiding damage to other pelvic organs [23]. Additionally, the rectangular‐shaped hemostatic sutures (a maximum of six sutures) play an important role in controlling and mitigating the massive bleeding before the uterine incision. Depending on the evaluation of the surgeon, uterine artery ligation for postpartum hemorrhage prevention could be added if necessary. However, the UAE was not used due to the high cost in low‐resource settings. According to the study of Munoz et al., intraoperative estimated blood loss of second‐trimester PAS delivery could vary between 1500 and 6750 mL (median: 2000–2600 mL). Among them, cesarean hysterectomy was highly observed [24]. Therefore, a proper surgical method should also be a great concern in the second‐trimester PAS management. In centers with available resource settings, elective robot‐assisted laparoscopic surgery could be performed in the early second‐trimester PAS [25, 26].

In general, rational management contributes significantly to reducing social, economic, and psychological repercussions for the pregnant individual [3, 10]. Furthermore, the pregnant women should be sufficiently informed about the risk of PAS recurrence, cesarean scar pregnancy, and massive postpartum hemorrhage in subsequent pregnancies if conservative uterine surgery is opted [27].

## 4. Conclusion

In conclusion, the DARIP procedure may be a feasible technique in managing the PAS during the second trimester of pregnancy. Particularly, this surgical approach could be achieved in a limited‐resource setting. However, a multidisciplinary team in an expert hospital should be sought in the management of PAS cases to optimize the patient′s outcomes. Further studies with a larger number of patients are essential to standardize the DARIP technique in clinical practice.

## Author Contributions

Ngoc Hai Tran and Van Hoang Bui were responsible for the conceptualization, methodology, investigation, administrative procedures, and supervision. Phuc Nhon Nguyen contributed to writing—review and editing and revised the manuscript.

## Funding

No funding was received for this manuscript.

## Disclosure

All authors read and approved the final manuscript. Phuc Nhon Nguyen is the guarantor of this study.

## Ethics Statement

The study was exempt from our Institutional Review Board approval for case reports at Tu Du Hospital. The study was performed in accordance with the ethical standards as laid down in the 1964 Declaration of Helsinki and its later amendments or comparable ethical standards.

## Consent

Written informed consent was obtained from the patient for the publication of this case report and accompanying images. A copy of the written consent is available for review by the editor‐in‐chief of this journal on request.

## Conflicts of Interest

The authors declare no conflicts of interest.

## Supporting Information

Additional supporting information can be found online in the Supporting Information section.

## Supporting information


**Supporting Information** Video S1: The lacunae with turbulent flows within the placenta and the rail sign are visible.

**Supporting Information** Video S2: The placenta is located at the anterior wall of the uterus, covering the internal os. Vascular proliferation appears at the cervix.

**Supporting Information** Video S3: Bridging vessels are observed at the vesicouterine interface.

**Supporting Information** Video S4: Three‐dimensional ultrasound shows the neovascularization signs inside the placenta.

**Supporting Information** Video S5: The DARIP technique.

## Data Availability

The datasets used and/or analyzed during the current study are available from the corresponding author upon reasonable request.

## References

[1] Jauniaux E. , Aplin J. D. , Fox K. A. , Afshar Y. , Hussein A. M. , Jones C. J. P. , and Burton G. J. , Placenta Accreta Spectrum, Nature Reviews Disease Primers. (2025) 11, no. 1, 10.1038/s41572-025-00624-3. 40473652

[2] Chen Q. , Shen K. , Wu Y. , Wei J. , Huang J. , and Pei C. , Advances in Prenatal Diagnosis of Placenta Accreta Spectrum, Medicina. (2025) 61, no. 3, 10.3390/medicina61030392, 40142202. PMC11943587 40142202

[3] Premkumar A. , Huysman B. , Cheng C. , Einerson B. D. , and Moayedi G. , Placenta Accreta Spectrum in the Second Trimester: A Clinical Conundrum in Procedural Abortion Care, American Journal of Obstetrics and Gynecology. (2025) 232, no. 1, 92–101, 10.1016/j.ajog.2024.07.045, 39117028. 39117028

[4] Yadav P. , Vatsa R. , Kulshrestha V. , and Malhotra N. , Conservative Management of Retained Placenta After Second-Trimester Termination of Pregnancy in Patients With Placenta Accreta Spectrum (PAS): Series of Three Cases, 2025, Journal of Obstetrics and Gynecology of India, 10.1007/s13224-025-02247-w.

[5] Harun M. H. H. , Ramli R. , Mohd Ali N. A. , and Abd Kadir N. J. , Placenta Accreta Spectrum Disorder Complicating a Second Trimester Miscarriage, BMJ Case Reports. (2025) 18, no. 2, e263686, 10.1136/bcr-2024-263686. 39933843

[6] Li Q. , Zhang W. , Hu C. , Zhao Y. , Pei C. , Wu X. , Fei K. , Peng Q. , Zhang J. , and Huang J. , Termination of a Second-Trimester Pregnancy With Placenta Accreta Spectrum Disorder, Libyan Journal of Medicine. (2023) 18, no. 1, 2258669, 10.1080/19932820.2023.2258669, 37722677. 37722677 PMC10512921

[7] Ho Q. N. , Tran N. H. , Vuong A. D. B. , Nguyen D. V. , Nguyen P. N. , and Nguyen Q. H. V. , Successful Management of Cesarean Scar Pregnancy Progressive to Placenta Accreta Spectrum: An Uncommon Condition in Vietnam and Mini-Review of the Literature, International Journal of Surgery Case Reports. (2025) 128, no. 128, 111076, 10.1016/j.ijscr.2025.111076, 40015227. 40015227 PMC11914175

[8] Taha O. T. , Abdelkarim M. , Al Qahtani N. , and Dawood A. S. , Shazly S. A. and Nassr A. A. , Management of Placenta Accreta Spectrum in the Second Trimester, Placenta Accreta Spectrum: Basic Science, Diagnosis, Classification and Management, 2023, Springer International Publishing, 185–192, 10.1007/978-3-031-10347-6_15.

[9] Kaba M. , Erkan C. , Sarica M. C. , and Mayir Y. A. , Emergency Hysterectomy After 2nd Trimester Abortion in a Patient With Placenta Accreta Spectrum Disorder Who Had Four Cesarean Deliveries, Ceska Gynekologie. (2023) 88, no. 2, 110–113, 10.48095/cccg2023110. 37130736

[10] Karunaratne M. S. , Matsuzaki S. , Matsushima K. , Yang J. L. , Mandelbaum R. S. , Muderspach L. I. , Ouzounian J. G. , Matsuo K. , and Nguyen B. T. , Obstetric Characteristics and Maternal Outcomes of Early Second-Trimester Placenta Accreta Spectrum, International Journal of Gynecology & Obstetrics. (2026) 172, no. 1, 622–629, 10.1002/ijgo.70372, 40693354. 40693354 PMC12724037

[11] Nieto-Calvache A. J. , Jauniaux E. , Fox K. A. , Maya J. , Stefanovic V. , Weizsäcker K. , van Beekhuizen H. , Adu-Bredu T. , Collins S. , Siaulys M. , Hussein A. M. , Duvekot J. , Aryananda R. , Pajkrt E. , Rijken M. J. , and the International Society for PAS (IS-PAS) LMIC Working Group , Are International Guideline Recommendations for the Management of Placenta Accreta Spectrum Applicable in Low- and Middle-Income Countries?, International Journal of Gynecology & Obstetrics. (2024) 166, no. 3, 1047–1056, 10.1002/ijgo.15473, 38488201. 38488201

[12] Thi Pham X. T. , Bao Vuong A. D. , Vuong L. N. , and Nguyen P. N. , A Novel Approach in the Management of Placenta Accreta Spectrum Disorders: A Single-Center Multidisciplinary Surgical Experience at Tu Du Hospital in Vietnam, Taiwanese Journal of Obstetrics and Gynecology. (2023) 62, no. 1, 22–30, 10.1016/j.tjog.2022.09.003, 36720545. 36720545

[13] Vuong A. D. B. , Pham T. H. , Pham X. T. T. , Truong D. P. , Nguyen X. T. , Trinh N. B. , Nguyen D. V. , Nguyen Y. O. N. , Nguyen T. N. T. N. , Ho Q. N. , and Nguyen P. N. , Modified One-Step Conservative Uterine Surgery (MOSCUS) Versus Cesarean Hysterectomy in the Management of Placenta Accreta Spectrum: A Single-Center Retrospective Analysis Based on 619 Vietnamese Pregnant Women, International Journal of Gynecology & Obstetrics. (2024) 165, no. 2, 723–736, 10.1002/ijgo.15220, 38009657. 38009657

[14] Bao Vuong A. D. , Thi Pham X. T. , and Nguyen P. N. , The Modified One-Step Conservative Uterine Surgery (MOSCUS) in the Management of Placenta Accreta Spectrum Disorders: Which, Where, When, and Who, Taiwan Journal Obstetrics Gynecology. (2023) 62, no. 4, 621–622, 10.1016/j.tjog.2023.04.008, 37407211. 37407211

[15] Jauniaux E. , Ayres-de-Campos D. , Langhoff-Roos J. , Fox K. A. , Collins S. , and FIGO Placenta Accreta Diagnosis and Management Expert Consensus Panel , FIGOclassification for the Clinical Diagnosis of Placenta Accreta Spectrum disorders, International Journal of Gynecology & Obstetrics. (2019) 146, no. 1, 20–24, 10.1002/ijgo.12761, 31173360. 31173360

[16] Walker S. P. , Bartels H. C. , Nieto-Calvache A. J. , Palacios-Jaraquemada J. M. , Collins S. L. , and Aryananda R. , Strategies for Streamlining Uterine Topographic Classification in Placenta Accreta Spectrum, AJOG Global Reports. (2026) 6, no. 2, 100636, 10.1016/j.xagr.2026.100636, 42064905. 42064905 PMC13126007

[17] Yu F. N. Y. and Leung K. Y. , Antenatal Diagnosis of Placenta Accreta Spectrum (PAS) Disorders, Best Practice & Research Clinical Obstetrics & Gynaecology. (2021) 72, no. 72, 13–24, 10.1016/j.bpobgyn.2020.06.010. 32747328

[18] Vuong A. D. B. , Nguyen X. T. , and Nguyen P. N. , Placenta Accreta Spectrum on an Unscarred Uterus in the Third-Trimester Pregnancy: Two Rare Cases at Tu Du Hospital in Vietnam, International Journal of Surgery Case Reports. (2022) 99, 107603, 10.1016/j.ijscr.2022.107603. 36150330 PMC9568723

[19] Nguyen V. H. , Huynh Q. H. , Ha T. N. , Nguyen M. C. N. , and Nguyen P. N. , Additional Role of Magnetic Resonance Imaging to Ultrasound in Assessing Placenta Accreta Spectrum Disorders: A Retrospective Cross-Sectional Study From Vietnam, Oman Medical Journal. (2024) 39, no. 6, 10.5001/omj.2024.119. PMC12010303 40260287

[20] Bucak M. , Chawla K. , Mark K. S. , Turan S. , and Turan O. M. , Standardized Algorithm for Cesarean Scar Pregnancy Management: Single-Center Outcomes, Journal of Maternal-Fetal & Neonatal Medicine. (2025) 38, no. 1, 2501693, 10.1080/14767058.2025.2501693, 40355381. 40355381

[21] Nguyen P. N. , Vuong A. D. B. , and Pham X. T. T. , Neonatal Outcomes in the Surgical Management of Placenta Accreta Spectrum Disorders: A Retrospective Single-Center Observational Study From 468 Vietnamese Pregnancies Beyond 28 Weeks of Gestation, BMC Pregnancy and Childbirth. (2024) 24, no. 1, 10.1186/s12884-024-06349-7, 38566074. PMC10986094 38566074

[22] Ou J. , Peng P. , Teng L. , Li C. , and Liu X. , Management of Patients With Placenta Accreta Spectrum Disorders Who Underwent Pregnancy Terminations in the Second Trimester: A Retrospective Study, European Journal of Obstetrics & Gynecology and Reproductive Biology. (2019) 242, no. 242, 109–113, 10.1016/j.ejogrb.2019.09.014, 31580962. 31580962

[23] Nguyen H. T. , Nguyen L. Q. , Truong P. T. , Nguyen P. T. , Hoang L. P. , and Nguyen P. N. , Rectangular-Shaped Hemostatic Sutures in the Management of Second-Trimester Placenta Accreta Spectrum Disorders at Tu Du Hospital, Vietnam: A Retrospective Descriptive Study, American Journal of Perinatology Reports. (2025) 15, no. 2, e79–e88, 10.1055/a-2608-0990. 40503449 PMC12151718

[24] Munoz J. L. , Counts R. , Lacue A. E. , Ireland K. E. , Ramsey P. S. , and Brandi K. , Surgical Outcomes and Associated Morbidity of Active and Expectant Management of Second-Trimester Placenta Accreta Spectrum (PAS), Medicina. (2025) 61, no. 1, 10.3390/medicina61010113, 39859095. PMC11766860 39859095

[25] Elfeky A. , Son M. A. , Paiva C. , and Alagkiozidis I. , Elective Robotic Hysterectomy for Placenta Accreta Spectrum in the Second Trimester: Case Report, International Journal of Surgery Case Reports. (2020) 12, no. 72, 361–364, 10.1016/j.ijscr.2020.06.024. PMC7306544 32563821

[26] Bahadur A. , Gattani S. , Singh G. , and Heda A. , Innovative Robotic Approach to Second-Trimester Placenta Accreta Spectrum Excision, Gynecology and Minimally Invasive Therapy. (2025) 14, no. 2, 193–194, 10.4103/gmit.GMIT-D-24-00014. 40521568 PMC12165669

[27] Zhao H. , Liu C. , Fu H. , Abeykoon S. D. I. , and Zhao X. , Subsequent Pregnancy Outcomes and Risk Factors Following Conservative Treatment for Placenta Accreta Spectrum: A Retrospective Cohort Study, American Journal of Obstetrics & Gynecology MFM. (2023) 5, no. 12, 10.1016/j.ajogmf.2023.101189, 101189, 37832645. 37832645

